# Effects of the glucagon-like peptide-1 receptor agonist liraglutide on retinal endothelial function and oxidative stress during sepsis

**DOI:** 10.3389/fphar.2026.1778848

**Published:** 2026-06-15

**Authors:** Elsa Wilma Böhm, Jenia Kouchek Zadeh, Wael Omran, Tschingis Arad, Norbert Pfeiffer, Felix M. Wagner, Andreas Patzak, Matthias Oelze, Andreas Daiber, Johanna Helmstädter, Sebastian Steven, Adrian Gericke

**Affiliations:** 1 Department of Ophthalmology, University Medical Center, Johannes Gutenberg -University Mainz, Mainz, Germany; 2 Institute of Translational Physiology, Charité-Universitätsmedizin Berlin, Berlin, Germany; 3 Laboratory of Molecular Cardiology, Department of Cardiology 1, University Medical Center of the Johannes Gutenberg-University, Mainz, Germany; 4 Department of Cardiology, University Heart Centre Frankfurt, University Hospital Frankfurt, Frankfurt, Germany

**Keywords:** endothelial dysfunction, glucagon like peptide, oxidative Stress, retina, sepsis

## Abstract

**Purpose:**

To test the hypothesis that the glucagon-like peptide-1 (GLP-1) receptor agonist liraglutide improves retinal vascular function in mice with polymicrobial sepsis.

**Methods:**

Three groups of mice were studied. Two groups underwent cecal ligation and puncture to induce polymicrobial sepsis. One received vehicle and the other liraglutide via intraperitoneal injection starting 24 h before the procedure and continuing twice daily until euthanasia. A third group underwent sham surgery and served as control. Forty-eight hours after sepsis induction, mice were euthanized and retinas were isolated for *ex vivo* assessment of vascular function by videomicroscopy. In addition, reactive oxygen species (ROS) formation and gene expression were evaluated using dihydroethidium staining, quantitative real-time PCR, and immunostaining of NADPH oxidase (NOX)1 and NOX2, respectively.

**Results:**

Endothelium-dependent vasodilation to acetylcholine was markedly impaired in retinal arterioles of septic, vehicle-treated mice but was partially preserved in liraglutide-treated septic mice. In contrast, vasodilation to the endothelium-independent vasodilator sodium nitroprusside was similar across all groups. Dihydroethidium staining revealed increased ROS signals in retinal arterioles, the ganglion cell layer, the inner and outer nuclear layers, and the optic nerve of septic, vehicle-treated mice. These increases were attenuated by liraglutide treatment. Furthermore, retinal mRNA expression of NOX1 was significantly upregulated in septic, vehicle-treated mice but remained at control levels in liraglutide-treated septic mice. . In addition, mRNA levels of SOD2, nNOS, and PAI-1 were increased, whereas NOX4, COX-2, and UCP-2 were decreased in septic, vehicle-treated mice. Liraglutide treatment was associated with increased mRNA expression for the antioxidant enzymes SOD1 and SOD3, reduced expression for MCP-1, ICAM-1 and PAI-1 mRNA levels and restoration of UCP-2 mRNA expression. Immunoreactivity for NOX1, but not NOX2, was increased in retinal blood vessels of septic mice.

**Conclusion:**

Treatment with liraglutide attenuates endothelial dysfunction, reduces oxidative stress, and suppresses NOX1 upregulation in the retina during polymicrobial sepsis. These findings highlight the preventive and therapeutic potential of GLP-1 receptor agonists for retinal vascular complications associated with sepsis and systemic inflammation.

## 1 Introduction

Sepsis is a leading global health burden and is estimated to account for nearly 20% of all deaths worldwide (Levy et al., 2012; Rudd et al., 2020). It arises from a dysregulated host response to infection characterized by excessive immune activation, which drives systemic inflammation, microvascular dysfunction, and ultimately life-threatening organ failure (Huang et al., 2019). In addition to hyperinflammation, secondary immune suppression, mitochondrial injury, oxidative stress, and coagulopathy further contribute to the progression of multiorgan dysfunction (Huang et al., 2019). Endothelial and microvascular impairment promote ischemic organ damage and microthrombotic events (Anas et al., 2010). Importantly, microvascular dysfunction has emerged as a prognostic indicator, as rapid restoration of microvascular perfusion in the initial phase of septic shock is observed in survivors of septic shock but not in non-survivors (Sakr et al., 2004). Moreover, microvascular injury contributes to long-term sequelae, including elevated cardiovascular risk—such as myocardial infarction, stroke, and fatal coronary heart disease—and persistent physical or cognitive impairments (Shankar-Hari and Rubenfeld, 2016).

Microvascular complications have also been reported in the eyes of patients with sepsis. Occlusion of retinal arteries, veins, or posterior ciliary arteries can lead to severe visual impairment (Aldrees and Micieli, 2020; Acharya et al., 2020; Damani et al., 2023; Raj et al., 2021; Vosoughi and Micieli, 2023). Experimental data from a porcine model of acute respiratory distress syndrome (ARDS) demonstrated impaired endothelial function in retinal arterioles, with oxidative stress identified as a mechanism, supported by elevated levels of reactive oxygen species (ROS) and increased expression of pro-oxidative enzymes such as NADPH oxidases (NOX) (Zadeh et al., 2019). NOX represent a major source of vascular ROS and are composed of different isoforms. While activation of NOX1 and NOX2 requires cytosolic components and activation by subunits, NOX4 is constitutively active. NOX1 and NOX2 are both expressed by vascular smooth muscle and endothelial cells and implicated in endothelial dysfunction, as well as promotion of vascular inflammation. As a consequence NOX1 and NOX2 are both associated with vascular disease by reducing nitric oxide (NO) bioavailability and the generation of harmful ROS, while protective functions of the NOX4 isoform have been described (Drummond et al., 2014; Konior et al., 2014).

Therapeutic approaches targeting microvascular dysfunction and oxidative stress during sepsis remain limited. Glucagon-like peptide-1 (GLP-1) receptor agonists have shown promise by improving endothelial function and exerting anti-inflammatory and antioxidant effects in both clinical and experimental sepsis (Yang et al., 2021; Helmstädter et al., 2021). Although the GLP-1 receptor is mainly expressed in pancreatic tissue, it is also present in cardiovascular cells, including atrial cardiomyocytes and vascular smooth muscle cells (Richards et al., 2014). Additionally, GLP-1 receptor expression has been determined in retinal tissue, particularly in ganglion cell layer neurons and retinal vascular endothelial cells (Hebsgaard et al., 2018; He et al., 2024).

Previous investigations using the cecal ligation and puncture (CLP) model of polymicrobial sepsis have demonstrated reduced inflammation and oxidative stress, along with improved endothelial function in large- and medium-sized vessels, such as the aorta and the ophthalmic artery, following treatment with GLP-1 receptor agonists (Helmstädter et al., 2021; Böhm et al., 2025). These findings indicate that GLP-1 receptor agonists modulate endothelial pathways as well as inflammation and oxidative stress across different vascular beds by activation of cAMP/protein kinase A (PKA)-related pathways with subsequent inhibition of NOX isoforms, upregulation of antioxidative enzymes and suppression of pro-inflammatory pathways (Oeseburg et al., 2010; Guo et al., 2015). The retina encompasses a unique autoregulated microvasculature characterized by high metabolic demand that is particularly vulnerable to inflammation and oxidative stress (Böhm et al., 2023). However, data on sepsis-induced alterations of the retinal microvasculature and on whether the protective mechanisms of GLP-1 receptor agonists extend to the retinal microcirculation remain limited. The CLP-procedure provides robust experimental model for investigating vascular pathophysiology and therapeutic interventions, including those targeting the ocular microvasculature.

Therefore, the present study aims to evaluate the preventive potential of the GLP-1 receptor agonist liraglutide, with a focus on endothelial dysfunction and oxidative stress in retinal arterioles as primary endpoint in a murine model of polymicrobial sepsis. By assessing microvascular function *ex vivo* using videomicroscopy and performing detailed localization of ROS within retinal tissue, this study provides novel insights into the deleterious effects of sepsis and the protective effects of GLP-1 receptor agonists on the retinal microvasculature.

## 2 Materials and methods

### 2.1 Animals

All experiments and treatments of animals were performed in accordance with the *Guide for the Care and Use of Laboratory Animals* (U.S. National Institutes of Health). Ethical approval was obtained in advance from the Ethics Committee of the University Hospital Mainz and the Landesuntersuchungsamt Rheinland-Pfalz (Koblenz, Germany; permit number: 23 177-07/G 14-1-039). The study was conducted in male C57BL/6J mice aged 8–12 weeks, all purchased from Charles River. The mice were housed in a 12-h light/dark cycle located in the institutional animal facility and had guaranteed free access to food and water.

Mice were randomly allocated to three groups: vehicle-treated sham-operated controls, vehicle-treated septic mice, and liraglutide-treated septic mice. Sepsis was induced by cecal ligation and puncture (CLP), a well-established model of polymicrobial sepsis characterized by bacteremia, excessive host immune activation, septic shock, and multiorgan failure (Rittirsch et al., 2009). Beginning 24 h before surgery, mice received intraperitoneal injections of liraglutide (0.1 mg/kg) or saline (vehicle, adequate volume for sham-operated and septic mice) twice daily at 12 h intervals until euthanasia with standardized dosing times relative to surgery. The CLP-procedure was performed as previously described (Helmstädter et al., 2021). In brief, mice were anesthetized by intraperitoneal injection of ketamine/xylazine (120 mg/kg ketamine and 16 mg/kg xylazine in 0.9% NaCl). Following a midline laparotomy, the cecum was exposed, ligated below the ileocecal valve, and punctured once with a 21-gauge needle. A small amount of fecal material was gently extruded into the peritoneal cavity to induce bacterial peritonitis. The cecum was then repositioned, and the abdominal musculature and skin were closed with running sutures. Postoperative fluid resuscitation consisted of prewarmed saline (37 °C, 50 mL/kg, s.c.). Sham-operated mice underwent identical surgical procedures except for cecal ligation and puncture. All surgeries were performed at the same time of day by the same operator to ensure consistency. Postoperative care included close monitoring and buprenorphine analgesia (0.05 mg/kg, s.c.) every 6 h. At 48 h after CLP, mice were euthanized by cardiac puncture under ketamine/xylazine anesthesia (120 mg/kg ketamine and 16 mg/kg xylazine in 0.9% NaCl).

Only male mice were included to ensure a controlled and internally consistent assessment of retinal microvascular responses in this experimental model, without introducing additional biological variables (Kong et al., 2015).

### 2.2 Preparation of murine retina and measurement of vascular reactivity

Following euthanasia, eyes with retrobulbar tissue were excised and immediately placed in cold Krebs buffer. Preparation of the retina and assessment of vascular reactivity in retinal arterioles by videomicroscopy were performed as reported previously (Gericke et al., 2018; Gericke et al., 2011). Briefly, under a dissection microscope, the ophthalmic artery and its side branches were carefully isolated from surrounding orbital tissue, and all side branches were ligated. To inactivate retrobulbar blood vessels, the intact eyeball was immersed in 70% ethanol for 10s and subsequently washed thoroughly in cold Krebs buffer. The eyeball was then opened by puncturing the cornea with a 30 G needle. The cornea and sclera were cut with Vannas scissors and gently removed to expose the retina. Next, the retina was transferred to an organ chamber containing cold Krebs buffer, and the ophthalmic artery was cannulated with a glass micropipette and secured using a 10–0 nylon monofilament suture. The retina was then positioned on a transparent plastic platform and fixed at the base with a steel ring. Retinal arterioles were pressurized to 50 mmHg through the micropipette placed in the ophthalmic artery via a Krebs-filled reservoir. During measurements, the organ chamber was continuously perfused with oxygenated and carbonated Krebs buffer maintained at 37 °C and pH 7.4. One single arteriole of each retina was visualized using a video camera attached to a microscope (Olympus Vanox-T AH-2; Olympus Deutschland GmbH, Hamburg, Germany). Blinding of investigators during *ex vivo* retinal arteriolar videomicroscopy was not feasible due to organizational constraints. After a 30-min equilibration period, first-order retinal arterioles were identified for functional analyses. Concentration-response curves to the thromboxane A_2_ receptor agonist U46619 (10^−11^ to 10^−6^ M; Cayman Chemical Company, MI, Germany) were first obtained. Following preconstriction of the same vessel segment to 50%–70% of its baseline diameter by titrating U46619, vasodilator responses to the endothelium-independent nitric oxide donor sodium nitroprusside (10^−9^ to 10^−4^ M, Sigma-Aldrich, Steinheim, Germany) and the endothelium-dependent vasodilator acetylcholine (10^−9^ to 10^−4^ M, Sigma-Aldrich) were recorded.

### 2.3 Quantification of reactive oxygen species

The fluorescent dye dihydroethidium (DHE) was used to quantify the overall formation of ROS in retinal layers and the optic nerve. Following euthanasia, mouse eyeballs were enucleated, embedded in Tissue-Tek O.C.T. Compound (Sakura Finetek Europe, Alphen aan den Rjin, Netherlands), and snap frozen in liquid nitrogen. Cryosections (10 μm) were prepared using a Leica Reichert Jung 2030 cryostat (Leica, Rijswijk, Netherlands) and mounted on SuperFrost Plus™ glass slides (Thermo Fisher Scientific, Waltham, MA, United States of America). The O.C.T. compound was removed by rinsing the sections in deionized water for 30 s. Tissue sections were then incubated with 1 μM DHE (Thermo Fisher Scientific, Waltham, MA, United States of America) for 30 min at 37 °C. Red fluorescence was visualized using an Eclipse TS 100 microscope (Nikon, Tokyo, Japan) equipped with a DS-Fi1-U2 digital microscope camera, an ELWD 20X/0.45 S Plan Fluor Ph1 ADM objective (Nikon), and the NIS Elements imaging software (Version 1.10, 64-bit; Nikon) with a TRITC filter setting. Fluorescence intensity was quantified by densitometry using ImageJ software (National Institutes of Health, Bethesda, MA, United States of America), as reported previously (Birk et al., 2021; Dauth et al., 2023). Regions of interest, including retinal layers, vascular structures and the optic nerve, were manually defined based on established anatomical landmarks on DHE-stained retinal and optic nerve cryosections and were consistently placed within these structures for densitometric analysis.

### 2.4 Quantitative PCR

Quantitative real-time PCR (qPCR) was conducted to determine mRNA levels of pro-oxidative enzymes (NOX1, NOX2, and NOX4), anti-oxidative enzymes (superoxide dismutase (SOD)1, SOD2, SOD3), nitric oxide synthases (NOS) (inducible NOS (iNOS), neuronal NOS (nNOS), endothelial NOS (eNOS)), cyclooxygenases (COX-1, COX-2), cytokines/adhesion molecules (monocyte chemoattractant protein (MCP)-1, MCP-2, intercellular adhesion molecule (ICAM)-1, vascular cell adhesion molecule (VCAM)-1) and regulatory proteins (plasminogen activator inhibitor (PAI)-1, uncoupling protein (UCP)-2) in isolated retinas from vehicle-treated sham-operated mice, vehicle-treated septic mice, and liraglutide-treated septic mice. Immediately after euthanasia, retinas were dissected using Vannas scissors and fine-point-tweezers, transferred to 1.5 mL plastic tubes, snap frozen in liquid nitrogen, and stored at −80 °C for 2–3 weeks until processing. Retinal tissue was homogenized in lysis buffer containing 1.0% NP40, 0.5% sodium deoxycholate, 0.1% SDS, 10 mmol/L NaF, and 80 mmol/L Tris (pH 7.5). For qPCR, a StepOnePlus system (Applied Biosystems, Foster City, CA, United States of America) was used. Cycling conditions were performed according to the manufacturers’ protocols: an initial denaturation step at 95 °C for 10 min; followed by 40 cycles of amplification at 95 °C for 20 s and 60 °C for 40 s; and a melt-curve analysis from 60 °C to 95 °C, increasing by 0.5 °C every 5 s. SYBR Green was used for fluorescence detection. Relative mRNA expression levels were calculated using the comparative threshold (CT) method, with normalization to β-actin (ACTB) as the reference gene. The primer sequences are presented in Table 1.

**TABLE 1:** Primer sequences used for quantitative PCR analysis in retinal samples.

Gene	Accession number	Forward	Reverse
*NOX1*	NM_172203.2	GGT​TGG​GGC​TGA​ACA​TTT​TTC	TCG​ACA​CAC​AGG​AAT​CAG​GAT
*NOX2*	NM_007807.5	GCA​CCT​GCA​GCC​TGC​CTG​AAT​T	TTG​TGT​GGA​TGG​CGG​TGT​GCA
*NOX4*	NM_015760.5	GGC​TGG​CCA​ACG​AAG​GGG​TTA​A	GAG​GCT​GCA​GTT​GAG​GTT​CAG​GAC​A
*SOD1*	NM_011434.1	CCA​GTG​CAG​GAC​CTC​ATT​TTA​AT	TCT​CCA​ACA​TGC​CTC​TCT​TCA​TC
*SOD2*	NM_013671	CCT​GCT​CTA​ATC​AGG​ACC​CAT​T	CGT​GCT​CCC​ACA​CGT​CAA​T
*SOD3*	NM_011435	TTC​TTG​TTC​TAC​GGC​TTG​CTA​CTG	AGC​TGG​ACT​CCC​CTG​GAT​TT
*eNOS*	NM_008713	CCT​TCC​GCT​ACC​AGC​CAG​A	CAG​AGA​TCT​TCA​CTG​CAT​TGG​CTA
*iNOS*	NM_010927.4	TTC​ACC​CAG​TTG​TGC​ATC​GAC​CTA	TCC​ATG​GTC​ACC​TCC​AAC​ACA​AGA
*nNOS*	NM_008712	TCCACCTGCCTCGAAACC	TTG​TCG​CTG​TTG​CCA​AAA​AC
*COX-1*	NM_008969.4	CCG​GAT​TGG​TGG​AGG​TAG​GAA​CTT​TGA​C	GGC​GCA​TCT​CTC​GGG​ACT​CCT​TG
*COX-2*	NM_011198.5	AGC​GAG​GAC​CTG​GGT​TCA​C	AAG​GCG​CAG​TTT​ATG​TTG​TCT​GT
*MCP-1*	NM_011333.3	TGA​TCC​CAA​TGA​GTA​GGC​TGG​AG	ATG​TCT​GGA​CCC​ATT​CCT​TCT​TG
*MCP-2*	NM_021443.3	AGT​GCT​TCT​TTG​CCT​GCT​GCT​CAT​AG	ATG​AGA​AAA​CAC​GCA​GCC​CAG​GCA​CC
*ICAM-1*	NM_010493.3	TTC​ACA​CTG​AAT​GCC​AGC​TC	GTC​TGC​TGA​GAC​CCC​TCT​TG
*VCAM-1*	NM_011693.3	GCC​CAC​TAA​ACG​CGA​AGG​T	ATG​GTC​AGA​ACG​GAC​TTG​GAC
*PAI-1*	NM_008871.2	CAC​AGG​CAC​TGC​AAA​AGG​TC	TTG​TCT​CTG​TCG​GGT​TGT​GC
*UCP-2*	NM_011671.6	ATC​GCC​TCC​CCT​GTT​GAT​GTG​GTC​A	CTC​AGA​AAG​GTG​CCT​CCC​GA
*ACTB*	NM_007393.5	CAC​CCG​CGA​GCA​CAG​CTT​CTT​T	AAT​ACA​GCC​CGG​GGA​GCA​TC

### 2.5 Immunostaining of the prooxidative enzymes NOX1 and NOX2

Retinal cryosections (10 µm thickness) were prepared and subjected to immunostaining. The sections were fixed in 4% paraformaldehyde for 20 min, followed by three washes in PBS (3 × 5 min). A blocking solution containing 0.1% Triton X-100% and 1% bovine serum albumin was applied for 30 min. Sections were then incubated for 2 h at room temperature with a primary antibody against NOX1 (ab131088, rabbit, Abcam, Waltham, MA, United States of America, dilution 1:200) and NOX2 (ab129068, rabbit, Abcam, dilution 1:200) diluted in blocking solution. In a prior study from our group, both antibodies induced marked staining in the aortic endothelium of apolipoprotein E–deficient mice, unlike in wild-type controls (Buonfiglio et al., 2023). Negative control sections were incubated with blocking solution only, without the primary antibody. Omission of the primary antibody resulted in an absence of detectable signal. After washing in PBS (3 × 5 min), sections were incubated for 1 h at room temperature with a Rhodamine Red-X–conjugated secondary antibody (111–295-003, goat anti-rabbit polyclonal, Dianova GmbH, Hamburg, Germany; dilution 1:200). Slides were subsequently rinsed in PBS, mounted with VECTASHIELD® Mounting Medium containing DAPI (BIOZOL Diagnostica Vertrieb GmbH, Eching, Germany), and coverslipped. All samples were processed under identical staining conditions to ensure comparability. Images were acquired using an Eclipse TS100 microscope (Nikon, Tokyo, Japan) equipped with a DS-Fi1-U2 digital camera and an ELWD 20×/0.45 S Plan Fluor Ph1 ADM objective (Nikon). NIS-Elements imaging software (Version 1.10, 64-bit; Nikon) was used with TRITC and DAPI filter settings to obtain representative images of retinal cryosections.

### 2.6 Statistical analysis

For comparison of vascular responses, two-way repeated measures ANOVA and Tukey’s multiple comparisons test were used. Data was tested for normal distribution using Shapiro–Wilk test prior to the selection of parametric or non-parametric statistical analyses. Fluorescence intensity was compared using one-way ANOVA and Tukey’s multiple comparisons test. Since mRNA expression data are not normally distributed, the Kruskal–Wallis test and the Dunn’s multiple comparisons test were applied, and data are presented as boxplots. The level of significance was set at p < 0.05. The value of n refers to the number of mice per experimental group. Statistical analysis was performed with GraphPad Prism 8 (GraphPad Software Inc., San Diego, CA, USA).

## 3 Results

### 3.1 Effects of liraglutide on retinal endothelial function in septic mice

**FIGURE 1:**
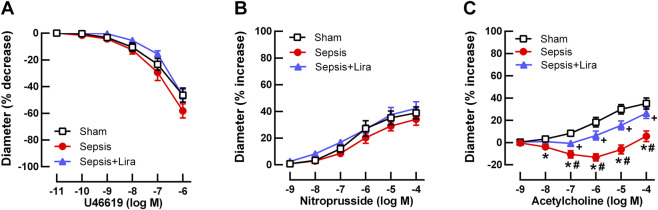
The thromboxane A_2_ mimetic U46619 caused concentration-dependent vasoconstriction of similar extent in vehicle-treated sham-operated (sham), vehicle-treated septic mice (sepsis) and liraglutide-treated septic mice (sepsis + lira) **(A)** Vasodilatory responses to the endothelium-independent vasodilator sodium nitroprusside **(B)** did also not differ between all three groups. Remarkably, responses to the endothelium-dependent vasodilator acetylcholine **(C)** were significantly impaired in vehicle-treated septic mice compared to vehicle-treated sham-operated mice. This endothelial dysfunction was partially prevented by treatment with liraglutide (*p < 0.05 sepsis vs. sham; ^#^p < 0.05 sepsis vs. sepsis + lira; ^+^p < 0.05 sepsis + lira vs. sham; n = 6 per group).

All three groups exhibited a concentration-dependent vasoconstrictor response to the thromboxane A_2_ receptor agonist U46619 (10^−11^–10^−6^ M), with no significant differences between vehicle-treated sham-operated mice, vehicle-treated septic mice, and liraglutide-treated septic mice (Figure 1A). Similarly, the endothelium-independent vasodilator sodium-nitroprusside (10^−9^–10^−4^ M) induced comparable concentration-dependent vasodilation across all groups (Figure 1B). In contrast, endothelium-dependent vasodilation to acetylcholine (10^−9^–10^−4^ M) was markedly impaired in vehicle-treated septic mice compared with vehicle-treated sham-operated mice, indicating pronounced endothelial dysfunction (p < 0.001, Figure 1C). Treatment with liraglutide partially preserved acetylcholine-induced vasodilation in septic mice (p < 0.001, Figure 1C).

### 3.2 ROS levels in retinal layers and the optic nerve

**FIGURE 2:**
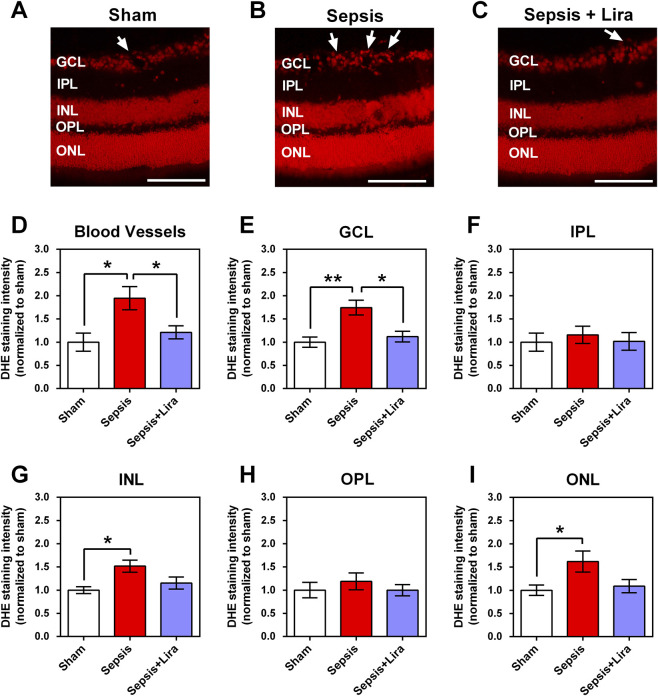
Representative images of DHE stainings of retinal cross-sections from vehicle treated sham-operated mice [sham, **(A)**] vehicle-treated septic mice [sepsis, **(B)**] and liraglutide-treated septic mice [sepsis + lira, **(C)**] Scale bar = 100 μm. Increased fluorescence intensity as a marker for ROS generation and oxidative stress was detected in retinal blood vessels **(D)** white arrows point on blood vessels), the ganglion cell layer [GCL, **(E)**] the inner nuclear layer [INL, **(G)**] and the outer nuclear layer [ONL, **(I)**] but not in the inner [IPL, **(F)**] and outer plexiform layers [OPL, **(H)**] of septic mice. This excessive ROS generation could be prevented by liraglutide (**p < 0.01; *p < 0.05, n = 6 per group).

**FIGURE 3:**
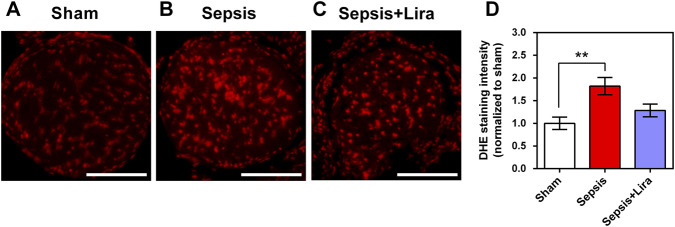
Representative pictures of DHE stainings of optic nerve cross-sections from vehicle treated sham-operated mice [sham, **(A)**] vehicle-treated septic mice [sepsis, **(B)**] and liraglutide-treated septic mice [sepsis + lira, **(C)**] Scale bar = 100 μm. Staining intensity was markedly increased in cross-sections of septic mice compared to those from sham-operated mice **(D)** Values are presented as mean ± SE (n = 6 per group; ^∗∗^p < 0.01).

ROS levels in individual retinal layers were quantified by measuring the fluorescence intensity of DHE-stained retinal cross-sections (Figures 2A-C). Retinal vessels from vehicle-treated septic mice exhibited a significant increase in DHE fluorescence compared with those from vehicle-treated sham-operated mice, indicating enhanced ROS generation during sepsis (Figure 2D). Liraglutide treatment markedly attenuated ROS formation in retinal vessels from septic mice (Figure 2D). Similar results were observed in the ganglion cell layer (GCL, Figure 2E), the inner nuclear layer (INL, Figure 2G), and the outer nuclear layer (ONL, Figure 2l), but not the inner (IPL, Figure 2F) and outer plexiform layers (OPL, Figure 2H). DHE staining intensity was also increased in optic nerve cross-sections of septic mice (Figure 3), suggesting that sepsis-induced oxidative stress extends to the ganglion cell axons.

### 3.3 Messenger RNA expression for redox and inflammatory markers

**FIGURE 4:**
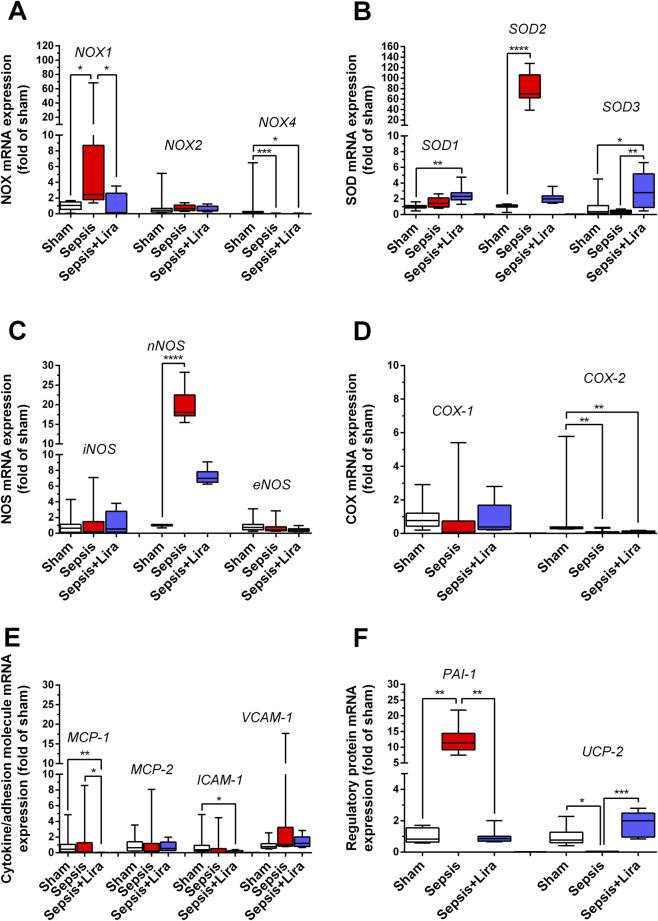
Messenger RNA expression for NOX **(A)** SOD **(B)** NOS **(C)** and COX isoforms as well as for **(D)** cytokines/adhesion molecules **(E)** and regulatory proteins **(F)** Data are presented as boxplots with minimum and maximum values (*p < 0.05, **p < 0.01, ***p < 0.001, ****p < 0.0001, n = 8 per group).

Sepsis did not affect expression levels of the *β-actin* gene, which we used as our reference gene. Median C_t_ values for *β-actin* mRNA expression were 18.39 (17.96–18.92), 18.58 (17.91–19.99), and 18.72 (18.37–19.25) in vehicle-treated sham-operated mice, vehicle-treated septic mice, and liraglutide-treated septic mice, respectively (p = 0.2645; Kruskal–Wallis test; n = 8 per group). Messenger RNA expression for the prooxidative enzyme NOX1 in whole-retina homogenates was significantly increased in vehicle-treated septic mice compared with vehicle-treated sham-operated mice (Figure 4A). Treatment of septic mice with liraglutide prevented retinal NOX1 mRNA overexpression, with expression levels no longer significantly different from those of sham-operated controls (Figure 4A). Notably, NOX2 mRNA expression did not differ among the experimental groups, and NOX4 mRNA expression was downregulated in both septic mouse groups (Figure 4A). Messenger RNA expression for the antioxidative enzymes SOD1 and SOD3 was significantly upregulated in liraglutide-treated septic mice compared to vehicle-treated sham-operated mice (Figure 4B). In contrast, *SOD2* mRNA was significantly upregulated in vehicle-treated septic mice, but not in liraglutide-treated septic mice (Figure 4B). We found no changes in mRNA expression for iNOS and eNOS, but a significant upregulation of *nNOS* mRNA in vehicle-treated septic mice compared to vehicle-treated sham-operated mice (Figure 4C). Messenger RNA levels of COX-2 were significantly lower in both septic groups compared to the sham group, while no changes in mRNA levels of COX-1 were detected (Figure 4D). Likewise, mRNA levels for the cytokine MCP-1 and the cellular adhesion molecule ICAM-1 were significantly reduced in liraglutide-treated septic mice compared to the sham group (Figure 4E). Notably, mRNA expression of the regulatory protein PAI-1 was significantly elevated in vehicle-treated septic mice compared to vehicle-treated sham-operated mice and liraglutide-treated septic mice, while mRNA levels UCP-2 were significantly lower in vehicle-treated septic mice and retained in liraglutide-treated septic mice (Figure 4F).

### 3.4 NOX1 and NOX2 expression in the retina

**FIGURE 5:**
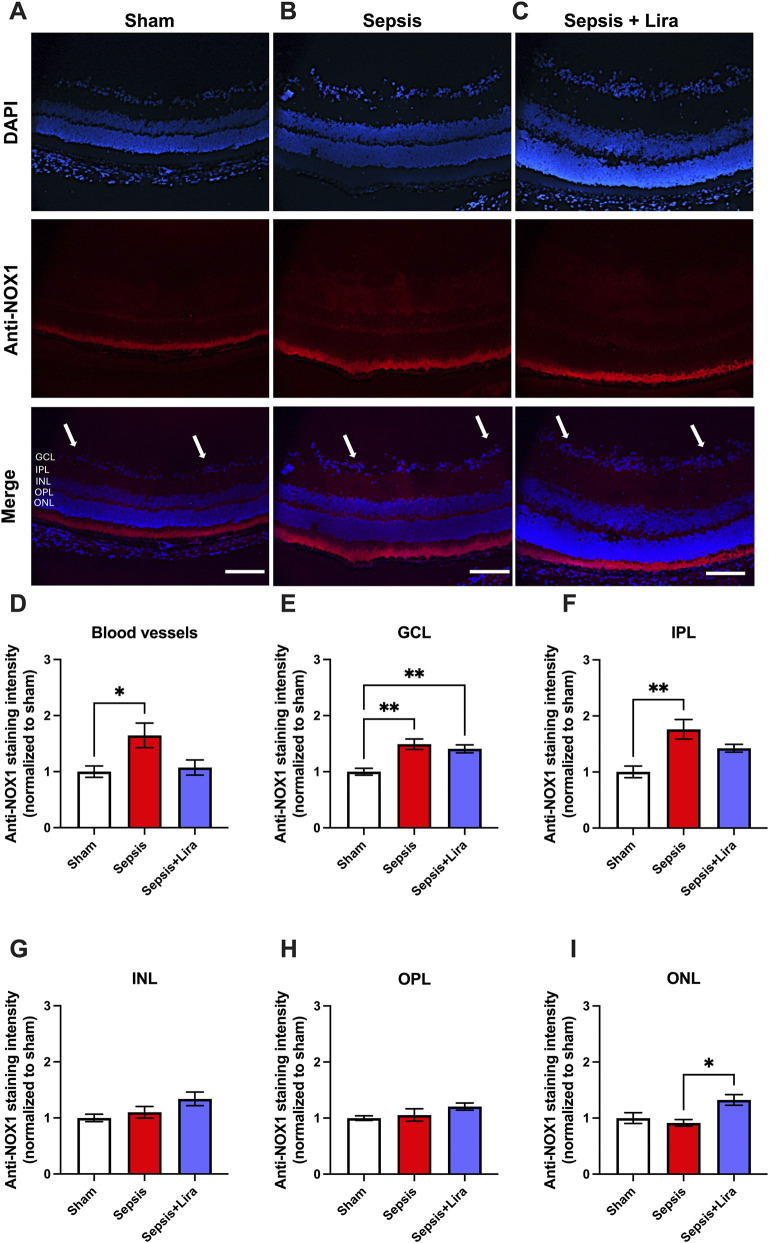
Representative images of anti-NOX1 stainings of retinal cross-sections from vehicle-treated sham-operated mice [sham, **(A)**] vehicle-treated septic mice [sepsis, **(B)**] and liraglutide-treated septic mice [sepsis + lira, **(C)**] Scale bar = 100 μm. Increased immunoreactivity was detected in retinal blood vessels [**(D)** white arrows point to blood vessels], the ganglion cell layer [GCL, **(E)**] and the inner plexiform layer [IPL, **(F)**] but not in the inner nuclear layer [INL, **(G)**] and outer plexiform layer [OPL, **(H)**] of septic mice. NOX1 immunoreactivity was significantly increased in the outer nuclear layer [ONL, **(I)**] of liraglutide-treated septic mice compared to septic mice (**p < 0.01; *p < 0.05, n = 6 per group).

**FIGURE 6:**
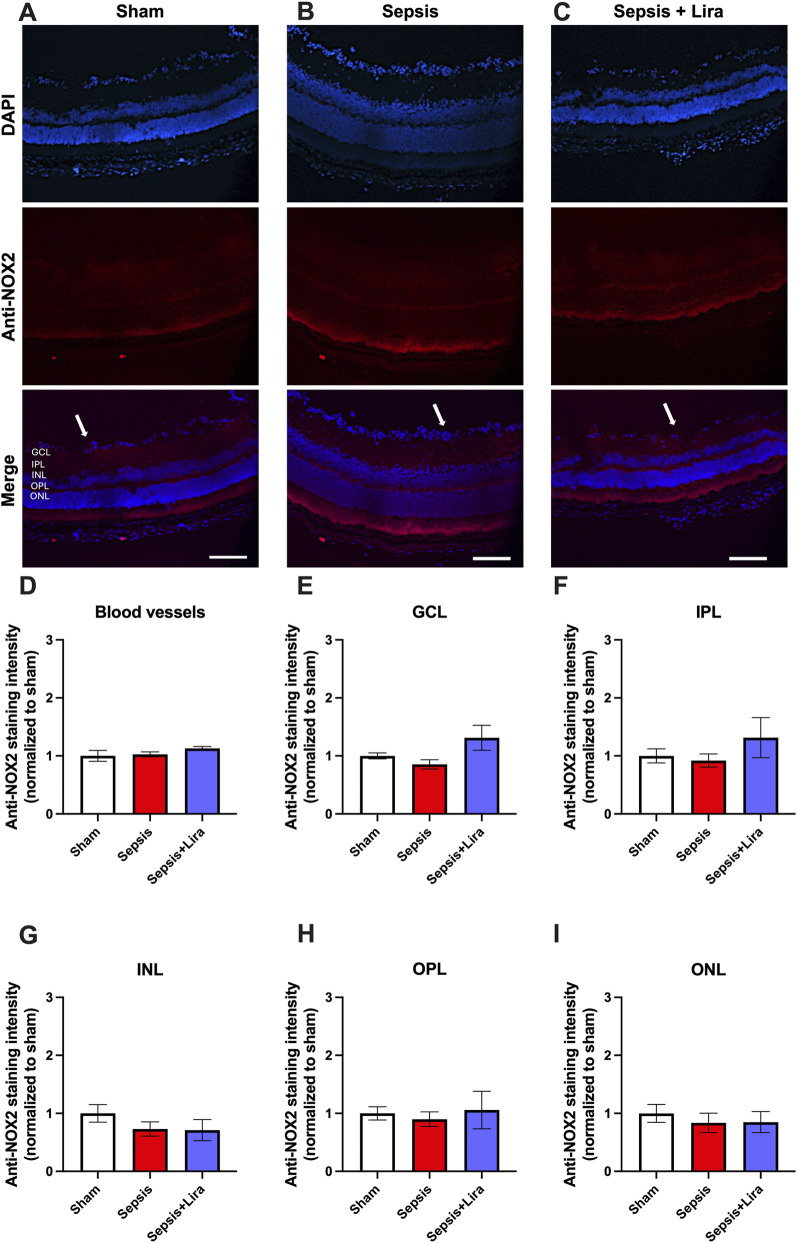
Representative images of anti-NOX2 stainings of retinal cross-sections from vehicle-treated sham-operated mice [sham, **(A)**] vehicle-treated septic mice [sepsis, **(B)**] and liraglutide-treated septic mice [sepsis + lira, **(C)**] Scale bar = 100 μm. No differences in immunoreactivity were observed in retinal blood vessels [**(D)** white arrows indicate blood vessels] or across retinal layers **(E–I)** among all groups (p > 0.05, n = 6 per group).

Immunostaining for the pro-oxidative enzymes NOX1 and NOX2 was performed on retinal cross-sections to assess protein expression. NOX1 immunoreactivity was significantly increased in retinal blood vessels of septic mice compared with the sham group. Treatment with the GLP-1 receptor agonist liraglutide led to a slight reduction in NOX1 expression in retinal blood vessels. However, this effect did not reach statistical significance (Figure 5D). A similar pattern was observed in the inner retinal layers, including the GCL and IPL (Figures 5E,F). No differences in NOX1 staining intensity were detected in the INL and OPL among the three groups (Figures 5G,H). In the ONL, NOX1 expression was significantly increased in liraglutide-treated septic mice compared with untreated septic mice (Figure 5I). No differences in NOX2 protein expression were observed in retinal blood vessels or across retinal layers among all groups (Figure 6). Representative images of anti-NOX1-and anti-NOX2-stained retinal cross-sections are shown in Figures 5A–C and Figures 6A–C.

## 4 Discussion

This study provides several important insights into the preventive and therapeutic potential of the GLP-1 receptor agonist liraglutide in mitigating endothelial dysfunction during polymicrobial sepsis. In our mouse model, sepsis markedly impaired endothelium-dependent vasodilation in retinal arterioles, indicating pronounced sepsis-induced endothelial dysfunction. Notably, treatment with liraglutide partially preserved endothelial function in septic mice. Our further analyses identified increased overall ROS generation in retinal arterioles, the GCL, the INL, and ONL, but not in the IPL, and OPL in septic mice. This increase was attenuated by liraglutide treatment. In retinal explants and retinal cross-sections, we also observed elevated expression of the pro-oxidative enzyme NOX1, suggesting its contribution to oxidative stress during sepsis. These indicators of oxidative stress were significantly ameliorated by liraglutide, suggesting that the compound exerts protective effects, at least in part, through the reduction of oxidative stress within the retinal vasculature. In the retina of septic mice, mRNA expression of SOD2, nNOS, and PAI-1 was significantly upregulated, whereas NOX4, COX-2, and UCP-2 were downregulated. Interestingly, liraglutide treatment resulted in upregulation of mRNA for the antioxidant enzymes SOD1 and SOD3, downregulation of retinal mRNA levels of MCP-1, ICAM-1 and PAI-1, and upregulation of UCP-2 mRNA levels, consistent with anti-oxidative and anti-inflammatory effects of liraglutide. Taken together, these findings suggest that GLP-1 receptor agonists may represent a promising preventive, but also therapeutic target for preventing retinal oxidative stress and endothelial dysfunction during polymicrobial sepsis.

Endothelial dysfunction is considered a critical step in the development of irreversible vascular damage, as it disrupts the balance of vasodilatory, anti-inflammatory, and antithrombotic functions of the endothelium. Accordingly, it represents an early marker of vascular disease, often preceding overt structural alterations and ultimately contributing to complications and end-organ damage (Poredos et al., 2021). While endothelial dysfunction in large and medium-sized vessels during sepsis has been extensively studied, little is known about endothelial function in the retinal microvasculature (Helmstädter et al., 2021; Bonjorno Junior et al., 2019). We previously demonstrated that sepsis induces endothelial dysfunction in the ophthalmic artery, a small-caliber vessel supplying the ocular circulation. In that study, endothelial function in the ophthalmic artery of septic mice was improved by treatment with liraglutide (Böhm et al., 2025). As the effects of sepsis on the retinal microcirculation remain poorly understood, we employed *ex vivo* videomicroscopy to assess responses in retinal arterioles. This technique enables detection of diameter changes as small as 1%–3% in microvessels of small laboratory animals (Böhm et al., 2022). Consistent with our findings in the ophthalmic artery, endothelial function was impaired in retinal arterioles of septic mice and was partially preserved by liraglutide treatment. To date, experimental studies that functionally assess endothelial dysfunction and investigate the underlying signaling pathways and specific sources of ROS in ocular tissues during systemic infection remain scarce. The observation of retinal endothelial dysfunction is novel and highly relevant, since ocular complications involving vascular occlusion, such as retinal vein and artery occlusion, or ischemic optic neuropathy, can result in profound visual impairment. Given that the retina and optic nerve are part of the central nervous system, microvascular alterations in ocular structures have also been associated with cognitive decline and the progression of central nervous system microvascular disease, contributing to an increased risk of stroke (Wong et al., 2001; Baker et al., 2007; Hanff et al., 2014). Therefore, a deeper understanding of the pathophysiological mechanisms driving endothelial dysfunction in sepsis is urgently needed to support the development of targeted therapeutic interventions aimed at improving both cardiovascular and ocular outcomes in patients recovering from sepsis.

Remarkably, we observed partially retained endothelial function in septic mice that were treated with liraglutide. This preventive and therapeutic potential of GLP-1 receptor agonists on retinal vascular function during septic conditions has been largely unexplored so far. Improvement of vascular endothelial function in other vascular beds by treatment with GLP-1 receptor agonists has been described in various cardiovascular disorders, including arterial hypertension, as well as in experimental models of sepsis (Helmstädter et al., 2021; Helmstädter et al., 2020). Additional preclinical studies investigating the mechanisms underlying the vascular effects of GLP-1 analogs have identified glucose-independent anti-inflammatory and anti-oxidative pathways, further supporting their potential to ameliorate endothelial dysfunction (Helmstädter et al., 2020; Kröller-Schön et al., 2012; Lee and June 2016). Using the same mouse cohort and study design as in the present study, liraglutide treatment restored aortic endothelial function in septic mice. Polymicrobial sepsis was associated with significantly reduced non-fasting blood glucose levels compared with non-septic controls. Importantly, liraglutide did not modify non-fasting glucose levels in septic mice, indicating potential glucose-independent effects of GLP-1 receptor agonists (Helmstädter et al., 2021). Likewise, sepsis-induced body weight loss was not influenced by liraglutide treatment. In contrast, liraglutide-treated septic mice exhibited normalized body temperature, as well as restored white blood cell and platelet counts, suggesting direct anti-inflammatory effects mediated by GLP-1 receptor activation (Helmstädter et al., 2021). Previous studies from our group have shown that liraglutide does not improve vascular endothelial function in septic mice lacking the GLP-1 receptor, indicating that its vasoprotective effects are largely receptor-mediated (Steven et al., 2017). However, direct GLP-1 receptor-dependent effects in the retinal vasculature remain to be confirmed.

Oxidative stress is a major contributor to endothelial dysfunction. Under physiological conditions, endothelium-dependent vasodilatation in mouse retinal arterioles is mainly mediated by eNOS (Gericke et al., 2013; Gericke and Buonfiglio, 2024). In the present study, endothelial function was assessed using acetylcholine. Acetylcholine stimulates endothelial M_3_ muscarinic receptors in retinal arterioles, leading to activation of eNOS and subsequent NO production (Gericke et al., 2011; Gericke et al., 2013). NO then diffuses to adjacent vascular smooth muscle cells, where it induces vasodilation (Daiber et al., 2017). Excessive production of ROS reduces NO bioavailability through its direct scavenging by superoxide, oxidation of tetrahydrobiopterin, the essential cofactor of eNOS, and in the next step eNOS uncoupling, thereby initiating a vicious circle of further ROS generation (Radi, 2018). Previous studies have consistently reported elevated ROS levels and oxidative stress markers during sepsis, especially in the context of endothelial dysfunction in various vascular beds (Huet et al., 2011; Zhang et al., 2025). However, to date, no studies have specifically assessed ROS levels in retinal tissue during sepsis. In the present study, we demonstrated increased overall ROS levels in retinal arterioles, the GCL, INL, ONL, and the optic nerve of septic mice, whereas no significant increase was observed in the IPL or OPL. These regional differences in oxidative stress may reflect variations in metabolic activity, mitochondrial density, vascular supply, and intrinsic antioxidant defense mechanisms. Retinal arterioles and the GCL are particularly metabolically active and mitochondria-rich, rendering them more susceptible to ROS generation (Gu et al., 2022). Similarly, in contrast to the IPL and OPL, the INL and ONL contain neuronal cell bodies with high metabolic demand, which may increase their vulnerability to oxidative stress. However, interpretation of DHE staining in plexiform layers is challenging, as the IPL and OPL predominantly consist of synaptic terminals, axons, and dendrites, preventing precise attribution of ROS signals to specific cell types. Notably, liraglutide treatment normalized retinal ROS levels in septic mice, suggesting antioxidative properties of this GLP-1 receptor agonist. Antioxidant effects of GLP-1 receptor agonists on retinal cells have not been reported in the context of sepsis-induced oxidative stress (Hou et al., 2024).

Our mRNA expression data and immunostaining results suggest that NOX1 may serve as a key source of ROS in the retina of septic mice, an effect that was attenuated by liraglutide treatment. NOX1 is recognized as a major contributor to retinal endothelial dysfunction across various disease models, representing a significant source of ROS under pathological conditions (Wilkinson-Berka et al., 2014; Deliyanti et al., 2020; Kaur et al., 2025). Downstream NOX1 signaling has been linked to disruption of retinal endothelial tight junctions and breakdown of blood-retinal barrier integrity (Kaur et al., 2025). In the ischemic retina, which may also occur during sepsis, NOX1 inhibition reduces leukocyte adhesion to the endothelium and decreases vascular inflammation and leakage (Deliyanti and Wilkinson-Berka, 2015). Although functional studies directly assessing the role of NOX1 in retinal endothelial dysfunction are lacking, studies in mesenteric arteries have shown that NOX1 knockout or selective pharmacological inhibition can restore endothelial vasodilatory function under pathological conditions (Padgett et al., 2026), highlighting the potential contribution of NOX1-derived ROS to endothelial dysfunction. Moreover, sepsis has been shown to activate NOX1-mediated ROS generation in human vascular endothelial cells, promoting vascular injury (Miyoshi et al., 2010). While downregulation of NOX1 by GLP-1 receptor agonists in retinal tissue has not been reported, evidence from other vascular beds and disease models suggests that GLP-1 receptor agonists can reduce vascular damage by suppressing NOX1 mRNA expression (Sukumaran et al., 2020; Marcondes-de-Castro et al., 2026). In the present study, NOX1 immunoreactivity was predominantly increased in the inner retinal layers, particularly in retinal arterioles, the GCL and IPL, during sepsis. This observation is consistent with the high metabolic activity and vulnerability of retinal ganglion cells and associated neurons, to inflammatory and ischemic stress, which are both characteristics of sepsis. Retinal ganglion cells have been reported to express NOX1 and its regulatory subunits under physiological and pathological conditions and may therefore represent an important source of ROS during inflammatory stress (Dvoriantchikova et al., 2012). The IPL mainly consists of synaptic connections between retinal ganglion cells and amacrine cells, and is thereby also susceptible to ischemic and inflammatory stress, contributing to increased NOX1 expression and redox activity within the inner retina (Wilkinson-Berka et al., 2014; Dionysopoulou et al., 2020). In contrast, outer retinal layers such as the INL and OPL showed no differences in NOX1 staining, which may reflect lower vascularization and metabolic activity, more robust antioxidant defenses, lack of the cellular machinery for NOX1 upregulation to septic stimuli, or differences in NOX isoform distribution. Furthermore, cell types of inner retinal layers, such as microglial and vascular cells have been shown to be more reactive to systemic inflammation and hypoxia thereby contributing to NOX1-associated ROS formation during sepsis. Microglia-derived oxidative stress has also been shown to be a major contributor to the pathogenesis of endothelial dysfunction (Wilkinson-Berka et al., 2014; Xiao et al., 2024). Collectively, these findings support the concept that sepsis preferentially affects the inner retinal neurovascular unit, where increased NOX1 expression may contribute to oxidative stress–related endothelial dysfunction. However, a cell type-specific attribution cannot be made based on the present immunohistochemical analysis alone.

While we previously reported pronounced upregulation of NOX2 mRNA and protein expression in ophthalmic arteries of vehicle-treated septic mice (Böhm et al., 2025), the present study did not provide evidence for increased NOX2 expression in retinal arterioles under septic conditions. In contrast, NOX1 emerged as the predominantly upregulated NOX isoform in retinal blood vessels. This vessel bed-specific divergence may be attributable to differences in local inflammatory signaling, leukocyte-endothelial interactions, and hemodynamic shear stress patterns, all of which are known to differentially regulate NOX1 and NOX2 activation (Drummond et al., 2014; Miyoshi et al., 2010; Siu et al., 2016). Notably, in a previous study in pigs from our laboratory, in which acute respiratory distress syndrome was induced by intratracheal administration of lipopolysaccharide, we observed endothelial dysfunction accompanied by marked upregulation of NOX2, but not NOX1, in retinal arterioles (Zadeh et al., 2019). However, several important differences between the studies should be considered, beyond the use of different species. First, the previous study employed an endotoxemia model associated with impaired pulmonary function and systemic hypoxia, whereas the present study is based on a polymicrobial sepsis model. Second, the experimental timelines differ substantially between these two studies. Measurements were performed 8 h after lipopolysaccharide administration in the previous study, compared with 48 h after sepsis induction in the current study. As no time-course analyses were conducted here, we cannot exclude the possibility that NOX2 predominates during the early, acute phase of sepsis, whereas NOX1 contributes to sustained or later-stage vascular stress (Knock, 2019).

Notably, we observed downregulation of NOX4 mRNA in septic mice and liraglutide-treated septic mice. Findings from other studies suggest that NOX4 is upregulated during septic conditions, but a potential protective role could also be identified during influenza A virus lung infections in mice, and downregulation of NOX4 expression was observed in virus-infected mice (Li J. et al., 2023; Hendricks et al., 2022). Overexpression of NOX4 leads to reduced lung inflammation and neutrophil infiltration as well as to a decreased oxidative burst from inflammatory cells compared to wildtype mice, especially in the early phase of sepsis (Hendricks et al., 2022). The protective effect declined after 7 days of infection (Hendricks et al., 2022). Thereby, the observed downregulation of NOX4 mRNA expression in whole-retina homogenates of our study might contribute to exacerbation of inflammation and oxidative stress, but up- or downregulation of the enzyme might be highly dependent on the phase of sepsis. In addition to NOX1, we also observed upregulated mRNA expression of the anti-oxidative SOD2 enzyme in septic mice, which may be a potential compensatory protective mechanism to attenuate oxidative stress and alleviate endothelial dysfunction. Such findings have also been reported in other studies (Constantino et al., 2014; Bao et al., 2022). This upregulation was weaker in retinas of septic, liraglutide-treated mice, which may suggest that this adaptive response is just attenuated following liraglutide treatment because the molecular deterioration is not as intense as in the vehicle-treated sepsis group. However, this observation is in contrast to non-septic oxidative stress models, where liraglutide has been shown to upregulate SOD2 and other antioxidant enzymes (Shiraki et al., 2012; Zhang et al., 2016). Hence, our results may reflect a reduced need for this compensatory response, as liraglutide attenuates upstream ROS generation through inhibition of NOX activity. However, it cannot be excluded that liraglutide may also directly suppress SOD2 induction, which could be detrimental if the antioxidant coverage provided by liraglutide is incomplete or transient. In contrast to SOD2, treatment of septic mice with liraglutide might also improve the anti-oxidant defense in our study cohort by upregulation of SOD1 and SOD3 expression in retinas of liraglutide-treated septic mice. These findings are in line with the current literature and underline the direct anti-oxidative effects and the high therapeutic potential of GLP-1 receptor agonists in vascular diseases associated with oxidative stress (Monji et al., 2013; Chang et al., 2015). SOD1 and SOD3 are main catalysators of the conversion of superoxide, a major ROS species implicated in endothelial dysfunction, into hydrogen peroxide. By subsequent improvement of NO bioavailability and reduction of eNOS uncoupling, that represent major steps in the pathogenesis of endothelial dysfunction during sepsis, endothelial function might be improved in liraglutide-treated septic mice. Given the differences of these antioxidative enzymes in their subcellular localization, these enzymes may be differentially involved in sepsis-related oxidative stress and may be differentially regulated by liraglutide treatment, what might explain our observations in mRNA expression (Faraci and Didion, 2004). During sepsis, expression of the mitochondrial inner membrane anion transporter UCP-2 is reduced. As UCP-2 normally limits mitochondrial ROS production by dissipating the electrochemical gradient, its downregulation contributes to enhanced inflammatory responses (Emre et al., 2007). Additionally, targeting UCP-2 was associated with improved endothelium-dependent vasodilation during endotoxemia (Toral et al., 2016). In our study, liraglutide treatment of septic mice could restore retinal mRNA expression levels of UCP-2, indicative of the potential of GLP-1 receptor agonists in attenuating mitochondrial ROS via targeting UCP-2. Since the retina is a highly metabolically active tissue, mitochondrial ROS might be a central mediator of sepsis-induced retinal endothelial injury. However, since our DHE staining results only reflect unspecific ROS generation, the specific role of mitochondrial ROS to sepsis-related oxidative stress and endothelial dysfunction in the retina and the role of GLP-1 receptor agonists in this scenario remains unclear and warrants future studies.

Inflammatory conditions such as sepsis lead to increased expression of pro-inflammatory genes, including TNF-α and iNOS, thereby enhancing oxidative stress and accelerating atherosclerotic processes (Kuhlencordt et al., 2001). TNF-α promotes iNOS expression, which subsequently increases ROS production, elevates inflammatory cytokine levels, upregulates vascular adhesion molecules, and contributes to endothelial dysfunction (Liu et al., 2023). In the vascular wall of CLP mice, sepsis induced significant iNOS upregulation, accompanied by increased mRNA levels of IL-6 and TNF-α, confirming a strong inflammatory state. Liraglutide treatment markedly reduced iNOS expression in this model (Helmstädter et al., 2021). In contrast, in the retinal tissue of our study, we observed increased mRNA levels of nNOS in septic mice, but not iNOS. Previous experimental studies identified nNOS-derived NO as a key player of pathological responses during sepsis, since inhibition of nNOS significantly reduced oxidative stress and inflammatory responses in septic sheep (Enkhbaatar et al., 2009). In contrast, another study reported that genetic deficiency and pharmacologic inhibition of nNOS in CLP-induced sepsis in mice, reduced survival, increased leukocyte trafficking to the infection site and decreased blood bacterial clearance (Cui et al., 2007). Upregulation of nNOS mRNA expression in our study might thereby either be a contributor to pathological effects of NO production or be a compensatory protective mechanism. Previous studies on vascular function of retinal arterioles also indicated that endothelial dysfunction with the lack of eNOS is compensated by nNOS as well as COX-2 metabolites (Gericke et al., 2019). We did not detect changes in mRNA expression of eNOS in whole retinal tissue of vehicle-treated, septic and liraglutide-treated, septic mice, suggesting that either eNOS expression was not altered under septic conditions or that changes in mRNA expression remained undetected, because eNOS is expressed only in a subset of cells in the retina (Gericke and Buonfiglio, 2024). The weakened nNOS upregulation by liraglutide may either indicate that eNOS function, and thus endothelium-derived NO production, is sufficiently preserved by liraglutide treatment, with subsequent reduction of the compensatory nNOS response or be a protective characteristic of liraglutide to reduce the harmful effects of NO during sepsis. But the suppression of this adaptive pathway warrants careful consideration and further investigation, especially with regard to studies of endothelial function.

Interestingly, COX-2, an inducible, vasoactive prostaglandine-producing enzyme, was downregulated in septic mice and also in liraglutide-treated septic mice. The fact of downregulation of COX-2 in both septic groups might more likely reflect that this effect is driven by sepsis *per se* rather than be liraglutide-associated. In studies of the central nervous system, COX-2 levels decreased in neuronal brain tissue during sepsis, while they were induced in perivascular tissue (Griton et al., 2020). Since the retina consists mainly of neuronal tissue, our results are in line with these findings, but the role of COX-2 on retinal endothelial function during sepsis warrants further studies. In our study, we could also identify potential anti-inflammatory effects of liraglutide, since downregulation of mRNA expression for the cytokine MCP-1, which is a key driver of monocyte recruitment during inflammation, and the cellular adhesion molecule ICAM-1, that enables binding and transmigration of immune cells to the vascular endothelium, was significantly reduced in liraglutide-treated septic mice compared to the vehicle-treated sham-operated group. However, since we did not find a significant upregulation of these genes during sepsis, it remains unclear whether these observations reflect a treatment-related modulation of inflammatory gene expression rather than a direct reversal of sepsis-induced inflammatory activation. Furthermore, we analyzed whole-retina homogenates, that do not allow direct conclusions on retinal vessels in the context of endothelial dysfunction. In addition, mRNA expression analysis was performed in whole-retina homogenates, which precludes cell type–specific attribution of these transcriptional changes to retinal endothelial cells, and their specific contribution to sepsis-associated endothelial dysfunction remains to be clarified. We found upregulation of PAI-1 mRNA expression in the retina of septic mice, that is known to be released by inflammatory stimuli and is associated with vascular endothelial dysfunction and promoted atherogenesis (Liu et al., 2009). PAI-1 has been identified as key driver of disseminated intravascular coagulation during sepsis and has been shown to be a predictive marker of mortality in septic patients (Li Y. et al., 2023). Within the retinal tissue, increased PAI-1 expression may thereby reflect inflammatory activation of the retinal microvasculature during sepsis, and may contribute to endothelial dysfunction, as observed in our study. However, PAI-1 has also been described to exert protective functions in the retina by limiting excitotoxicity-induced retinal damage (Mali et al., 2005). Thus, it remains unclear whether the observed upregulation of PAI-1 in septic mice of our study represents a marker of endothelial activation or a compensatory response to retinal injury. Notably, treatment with liraglutide could attenuate retinal upregulation of PAI-1 mRNA levels during sepsis, which might again reflect the vasoprotective potential of GLP-1 receptor agonists during sepsis.

A limitation of this study is that the mechanistic conclusions are primarily based on DHE-derived ROS measurements, which reflect overall oxidative stress rather than specific ROS species or cellular sources. Additionally, our mechanistic analyses relied largely on mRNA data, which do not establish causal redox pathways and should therefore be considered exploratory. The use of whole-retina homogenates for mRNA analysis further limits cell type–specific attribution of transcriptional changes. It also remains unclear whether the observed sepsis-induced endothelial dysfunction, and its preservation with liraglutide treatment, is mediated by changes in NO bioavailability, prostanoid signaling, or altered NOS activity. Future studies linking the observed functional vascular changes in retinal arterioles to our observed potential mechanistic findings based on mRNA and protein expression, by using inhibitors targeting NOX1 or NOS enzymes are warranted to fully characterize the mechanisms of retinal endothelial dysfunction during sepsis. Moreover, this study does not provide direct evidence of GLP-1 receptor–dependent effects, as the actions of liraglutide could involve off-target or systemic mechanisms. Future studies should validate our findings at the protein level and through functional assays. Moreover, because sepsis is a dynamic process, our study—conducted at a single time point and limited to male mice—may have restricted generalizability to other stages of sepsis or to female animals. Sex-specific differences in endothelial function, influenced by hormonal factors, are well recognized and may also be relevant in the context of sepsis and liraglutide treatment, warranting further investigation (Kong et al., 2015). Nevertheless, to our knowledge, this is the first study to demonstrate that liraglutide treatment is associated with preserved endothelial function and reduced oxidative stress in retinal arterioles in a mouse model of polymicrobial sepsis. Although further studies are required to elucidate the underlying molecular mechanisms, these findings suggest a potential protective role for GLP-1 receptor agonists in retinal microvascular dysfunction under pathological conditions.

## 5 Conclusion

This study demonstrates that CLP-induced polymicrobial sepsis leads to pronounced endothelial dysfunction in retinal arterioles. Increased oxidative stress, characterized by excessive ROS generation within the vascular wall, appears to be a key contributing mechanism. Upregulation of the pro-oxidative enzyme NOX1 in retinal tissue suggests that NOX1 might represent a possible source of ROS during sepsis. Notably, treatment with the GLP-1 receptor agonist liraglutide preserved endothelial function in septic mice, accompanied by reduced ROS formation and decreased mRNA expression of pro-oxidative enzymes. Furthermore, treatment with liraglutide restored alterations in retinal mRNA expression of the regulatory proteins PAI-1 und UCP-2 during sepsis. The potential anti-oxidant and anti-inflammatory capacities of GLP-1 receptor are further supported by upregulation of mRNA levels for the antioxidant enzymes SOD1 and SOD3, along with downregulation of mRNA expression for the inflammatory mediators MCP-1 and ICAM-1.

These findings underscore the vascular protective properties of the GLP-1 receptor agonist liraglutide beyond its glucose-lowering effect and highlight its potential as a therapeutic agent for conditions characterized by endothelial dysfunction, including polymicrobial sepsis.

## Data Availability

The original contributions presented in the study are included in the article/supplementary material, further inquiries can be directed to the corresponding author.
